# Effects of Microbial Activity and Environmental Parameters on the Degradation of Extracellular Environmental DNA from a Eutrophic Lake

**DOI:** 10.3390/ijerph16183339

**Published:** 2019-09-10

**Authors:** Nur Syahidah Zulkefli, Keon-Hee Kim, Soon-Jin Hwang

**Affiliations:** 1 Department of Environmental Health Science, Konkuk University, Seoul 05029, Korea; 2 Human & Eco-Care Center, Department of Environmental Health Science, Konkuk University, Seoul 05029, Korea

**Keywords:** extracellular DNA, degradation, biomonitoring, freshwater environment, microbial activity, temperature, light, pH

## Abstract

Extracellular DNA (exDNA) pool in aquatic environments is a valuable source for biomonitoring and bioassessment. However, degradation under particular environmental conditions can hamper exDNA detectability over time. In this study, we analyzed how different biotic and abiotic factors affect the degradation rate of extracellular environmental DNA using 16S rDNA sequences extracted from the sediment of a eutrophic lake and *Anabaena variabilis* cultured in the laboratory. We exposed the extracted exDNA to different levels of temperature, light, pH, and bacterial activity, and quantitatively analyzed the concentration of exDNA during 4 days. The solution containing bacteria for microbial activity treatment was obtained from the lake sediment using four consecutive steps of filtration; two mesh filters (100 μm and 60 μm mesh) and two glass fiber filters (2.7 μm and 1.2 μm pore-sized). We found that temperature individually and in combination with bacterial abundance had significant positive effects on the degradation of exDNA. The highest degradation rate was observed in samples exposed to high microbial activity, where exDNA was completely degraded within 1 day at a rate of 3.27 day^−1^. Light intensity and pH had no significant effects on degradation rate of exDNA. Our results indicate that degradation of exDNA in freshwater ecosystems is driven by the combination of both biotic and abiotic factors and it may occur very fast under particular conditions.

## 1. Introduction

A variety of organisms release their genetic material into the environment, which can be collected and analyzed to identify the presence and distribution of target organisms [1,2,3]. This genetic material is collectively termed environmental DNA (eDNA), whose detection allows us to trace the habitat use, selection, and occupancy of specific organisms [4,5,6]. Using water or sediment samples as eDNA sources, eDNA-based identification by molecular approach has shown potential to replace the traditional methods of biomonitoring and bioassessment in terms of improving the detectability of aquatic species [7,8,9].

Total eDNA consists of intracellular DNA (iDNA), which is contained in living cells or organisms, and extracellular DNA (exDNA), which is released because of cell lysis and death [10,11]. Even though iDNA and exDNA are simply distinguished based on whether the eDNA is located inside or outside cells at a given time, the fate of these two types of eDNAs can differ significantly. In the case of exDNA, the lack of a living cell to produce energy and support DNA replication, synthesis, and repair makes it more vulnerable to environmental influences [12]. Moreover, exDNA can be used by bacteria for natural transformation (e.g., horizontal gene transfer, HGT), and also as a nutrient source and biofilm matrix component [13,14,15].

The higher bacterial abundance in the sediment than in the water column [16,17,18] indicates that exDNA may be susceptible to potential biotic decay over time in natural ecosystem. The bacterial abundance found in the lakes of other studies ranged from 10^7^–10^9^ cells/g of wet sediment [17,19]. Accumulation of exDNA in the sediment provides nutrient sources to microbial community [11,20,21,22]. The sediment provides adsorbent properties via clay and humic acids binding to the exDNA for resistance against nucleases, and confers long persistence in the sediment [22]. The dissolved extracellular DNA (dDNA) collected from a stratified lake were found to persist longer in deeper water than in surface water, suggesting that dDNA can persist without degradation in the hypolimnion area and might be important as a genetic pool for natural transformation [23]. The released exDNA by aquatic organisms may be transported even deeper to be finally settled down with the sediment. This may explain why detection of DNA isolated from the sediment was often reported to be higher than those extracted from water samples [24,25].

The persistence of exDNA in the environment depends on various factors. It has been shown that sediment-associated exDNA persistence is affected by the interaction with environmental conditions in marine ecosystems [26]. Another study analyzed eDNA function through HGT and compared exDNA and iDNA persistence from both sediment and water samples. It was shown that exDNA persistence in the sediment is not just a by-product of iDNA conversion, but it is affected by environmental factors that may alter its stability [27]. Indeed, detectability of eDNA recovered from several aquatic organisms seems to vary with different environmental conditions. For instance, common carp was detected for approximately 4 days [28], bullfrog for 54 days [29], and bighead carp and silver carp for 14 days [30] using methods to assess iDNA fragments. Among other parameters tested, temperature and microbial activity were considered the main factors underlying eDNA degradation over time [28,29,30,31]. Acidity (pH) was also found to have a significant influence on eDNA degradation while light intensity seemed to play a minor role [29,32,33,34]. However, the interaction between various environmental parameters cannot be avoided in a real ecosystem, and might likely influence eDNA degradation depending on the parameter levels and interacting factors [29,35].

To our knowledge, previous studies were limited to the detection or degradation of exDNA pools in marine environments, evaluation of factors that contribute to the genetic composition, and characterization of taxa detected in field samples [26,36,37]. Moreover, although different environmental factors have been found to influence iDNA, the extent to which these factors affect the degradation of exDNA recovered from freshwater ecosystems remains unclear. For a better understanding which factors limit the detection of exDNA in freshwater environment, this study aimed to assess the effects of different environmental factors on the degradation rates of exDNA extracted from freshwater sediment samples. Therefore, we evaluated the effects of temperature, light intensity, pH, and microbial activity on exDNA detection and degradation rates over time. Based on the parameters, we hypothesized that degradation of exDNA occur rapidly in the lake sediment where microbial activity promoted by temperature is a main driving force. We also predicted that pH and light intensity might positively affect exDNA degradation when interacting with other factors. The findings of this study will provide new insights on exDNA degradation in freshwater environment such as eutrophic lake sediment, which could be useful for exDNA detection in the future studies.

## 2. Materials and Methods

### 2.1. Preparation of Extracellular eDNA Source

The sampling site was located at the littoral zone of a shallow eutrophic lake, Seoul, Korea (37°32′22″ N, 127°04′36″ E). The sediment samples were collected using a Petersen Grab Sampler (Q.T. Technology, Seoul, Korea), placed in a container and transported to the laboratory within 30 min, where they were used to isolate microorganisms and extract exDNA.

For exDNA extraction, a bulk of sediment samples taken from multiple grab samplings were combined and divided into equivalent 0.5 g aliquots and placed in NucleoSpin^®^ Bead Tube Type A, which contained ceramic beads included in NucleoSpin Soil Kit (Macherey-Nagel, Düren, Germany). This resulted in 87 sediment subsamples, which were used for exDNA extraction, following the bead-beating mechanical lysis and column-based DNA purification according to the extraction kit protocol [38,39]. The final elution of eDNA from the column was performed with 60 μL of elution buffer for each sample. Each aliquot of extracted exDNA was combined before determining the concentration.

We determined the concentration of exDNA extracted from sediment samples using a NanoDrop™ UV-Vis Spectrophotometer (Thermo Fisher Scientific, Waltham, MA, USA). Based on this first quantification, we detected that the total exDNA recovered from lake sediment would not be enough for the whole set of experiments. Therefore, we extracted exDNA from cyanobacteria, *Anabaena variabilis* AG-100064 (Korean Collection for Type Cultures, the Republic of Korea) to obtain additional DNA. The extraction of cyanobacterial DNA was performed using the same kit and methods described for the sediment samples. Then, we combined the DNA sample extracted from *A. variabilis* with previously extracted sediment DNA samples in a 1:1 ratio.

### 2.2. Preparation of Microbial Solution

We prepared bacterial solution by processing the same sediment samples of our extracted exDNA to observe the degradation by bacteria that naturally attached onto the target exDNA source. Sediment samples were mixed with 200 mL distilled water and serially filtered to obtain microorganisms for the microbial activity experiment. Through a consecutive filtration steps by using 100 μm and then 60 μm mesh filters, 200 mL of solution containing microorganisms were obtained. Then, the solution was divided into four 50 mL conical tubes in equivalent volume and centrifuged (Table Top Centrifuge VS-5000i, Vision Scientific Co., Daejeon, Korea) at 1000 rpm for 30 min at 4 °C. Then, the supernatant was discarded, distilled water was added up to the maximum volume of the 50-mL conical tubes and the solution was centrifuged once again. To remove any small algae or protists, the solution was consecutively filtered through 2.7-μm pore-sized GF/D and 1.2-μm pore-sized GF/C glass fiber filters (Whatman, Product No.: 1822-047 and 1823-047, respectively). Then, the final bacterial solutions were mixed together and divided into 3 tubes of equal volume representing different temperatures. Each sample was maintained according to its respective temperature (5 °C, 25 °C, or 35 °C) in a refrigerator or temperature-controlled incubators (Vision Scientific, VS-1203P4S, Daejeon, Korea) in the dark. Bacterial abundance in the solutions was 1.6 ± 0.12 × 10^7^ cells/mL or 3.2 ± 0.12 × 10^9^ cells/g of wet sediment, when counted using an epifluorescence microscope (Zeiss Axiostar plus, HBO 50/AC, Jena, Germany) after 4′6-diamidino-2-phenylindole (DAPI) staining protocol [40].

### 2.3. Experimental Setup

#### 2.3.1. Temperature Effect

We prepared 96-well microplates to expose exDNA samples to different temperatures (5 °C as control, 15 °C, 25 °C, and 35 °C), which were selected based on temperatures previously reported to be associated with DNA persistence and degradation [30,31] (Table 1). Each microplate well contained 50 μL of nuclease-free water and 50 μL of exDNA sample, making a total volume of 100 μL per treatment sample (*n* = 12). Experiments were run in triplicate. All samples were incubated for 4 days in the temperature-controlled incubators in the dark. A tray filled with distilled water was put in the incubators to avoid the evaporative loss of samples. An aliquot of 4 μL was taken from each treatment at 0, 24, 48, 72, and 96 h and used to quantify DNA using qPCR.

#### 2.3.2. pH Effect

The effect of pH on exDNA degradation was assessed under three different pH levels (4, 7, and 10) to represent a range of acidic, neutral (control), and alkaline water in temperate freshwater systems as suggested by Strickler et al. (2014). Acidic and alkaline conditions were adjusted with 1 M NaOH and 1 M HCl to reach the desired pH, whereas distilled water was used as the neutral pH treatment. To observe any combined effects of pH and temperature, we prepared two microplates containing each pH treatment to be incubated separately at either 5 °C or 35 °C (Table 1). This was done because we previously found that the lowest and the highest temperature had the most pronounced effects on exDNA persistence during the temperature experiment. In each microplate well, we added 50 μL of exDNA sample mixed with 50 μL of each solution at the desired pH, making a total volume of 100 μL per treatment sample. Each sample was assessed in triplicate (*n* = 18). An aliquot of 4 μL was taken from each treatment at 0, 24, 48, 72, and 96 h and used to quantify DNA using qPCR.

#### 2.3.3. Light Effect

The effect of light on exDNA degradation was assessed under different light intensity levels (20, 50, and 100 μmol m^−2^ s^−1^). One set of samples was kept in the dark as a control treatment. Fluorescence light bulbs were set up in incubators to get desired light intensities, which were confirmed by a portable hand-held Light Meter (Model LI-250A, LI-COR Biosciences, Lincoln, NE, USA). To observe any combined effects of light and temperature, we prepared two microplates to be incubated at either 5 °C or 35 °C for each light intensity (Table 1). In each microplate well, we added 50 μL of exDNA sample mixed with 50 μL of nuclease free water, making a total volume of 100 μL per treatment sample. Each sample was assessed in triplicate (*n* = 24). An aliquot of 4 μL was taken from each treatment at 0, 24, 48, 72, and 96 h and used to quantify DNA using qPCR.

#### 2.3.4. Microbial Effect

The effect of bacteria on exDNA degradation was assessed under three different temperatures (5 °C, 25 °C, and 35 °C). Each sample maintained in respective temperatures was treated with serial dilutions (10^0^, 10^−2^, and 10^−5^ fold) of solutions containing bacteria (average abundance: 1.6 ± 0.12 × 10^7^ cells/mL, or 3.2 ± 0.12 × 10^9^ cells/g of wet sediment) sourced from the sediment samples. A control group in which no bacteria was added was also assessed (Table 1). Each sample consisted of 50 μL of exDNA mixed with 50 μL bacterial solutions per treatment (*n* = 36). An aliquot of 4 μL was taken from each treatment at 0, 24, 48, 72, and 96 h and used to quantify DNA using qPCR.

### 2.4. Quantification of exDNA

ExDNA was quantified in the samples by qPCR using primers designed to detect cyanobacterial 16S rDNA sequences [41]: forward, 5′-CGGACGGGTGAGTAACGCGTG-3′, and reverse, 5′-CCCATTGCGGAAAATTCCCC-3′. Assays were run in a Rotor-Gene Q 2plex (Qiagen, Germantown, MD, USA). The PCR reaction mixture contained 10 μL of 2× Real-Time PCR Master Mix (BioFACT™ A-Star 2× Real-Time PCR kit (Biofact Co., Daejeon, Korea)) which includes the SYBR^®^ green, each primer at a concentration of 1 pM, 2 μL of template DNA, and distilled water adjusted to the final volume of 20 μL. The efficiency of qPCR was obtained at 99%. The qPCR reaction began with an initial activation reaction of 15 min at 95 °C, followed by 35 cycles comprising 5–15 s at 95 °C, 10–15 s at annealing temperature of 56 °C, 15–30 s at 72 °C. Then, final fluorescence was measured at 72 °C. Melting curve analyses were used to assess primer dimers. Least-square linear regression analyses of Ct values and gene copy numbers (N_P_) were calculated using the equation below (Equation (1)) [42] to determine the sample concentrations based on standard curves, where N_A_ is Avogadro constant, MW_bp_ is the average base pair molecular weight [43], and S_P_ is the genome size:

(1) $$N_{P}=\frac{N_{A}}{S_{P}\times \;{\mathrm{MW}}_{\mathrm{bp}}\;\times 10^{6}}$$

Fragmentation below the length of PCR fragment (258 bp) limited DNA detection by qPCR and indicated degradation. In this study, DNA degradation was defined by the decline of DNA concentration from the initial concentration assigned in the treatments over the duration of the experiment. DNA concentration was calculated as gene copy numbers using Ct values, which is determined by the number of cycles required for the fluorescence signal to cross a threshold recognized by the qPCR instrumentations. Since Ct levels are inversely proportional to the amount of target nucleic acid in the sample [44], degradation of target sequence is observed when Ct levels are delayed or below the adjusted threshold.

### 2.5. Statistical Analyses

All exDNA concentration data were analyzed to estimate any significant relationship between treatments and exDNA amount detected over the duration of the experiment. We applied log (x + 1) transformation to all exDNA data to achieve normality. One-way ANOVA was used to test for differences in exDNA degradation rates among the different levels of temperature, light intensity, pH, and microbial activity. The difference between samples was considered significant at *p* < 0.05.

We calculated the degradation rates of exDNA using the exponential decay model [N(t) = N_0_e^−*r*t^], which represents our data decay pattern. This pattern is similar to those observed in other studies [29,45]. In the decay model, N(t) is the concentration of exDNA at time t, N_0_ is the initial concentration of exDNA, and *r* is the degradation rate. All data obtained from the different treatments were fitted to standard curves created using SigmaPlot^®^ v. 10.0 (Systat Software Inc. (SSI), San Jose, CA, USA). All statistical analyses were run using PASW^®^ Statistic v. 18 (SPSS Inc., Chicago, IL, USA).

## 3. Results

### 3.1. Temperature Effect

**Figure 1:**
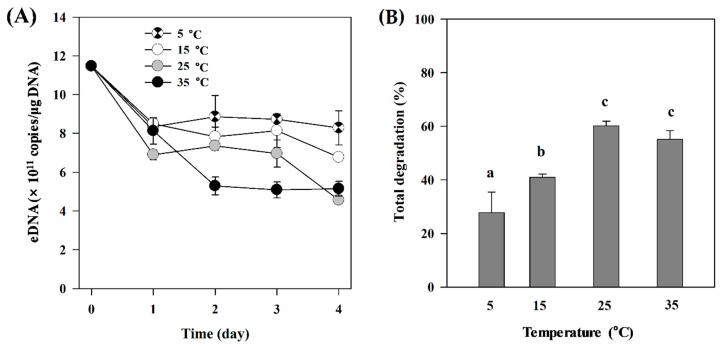
ExDNA concentration in samples exposed to different temperatures. (**A**) Changes in exDNA concentration detected under different temperature treatments (5 °C, 15 °C, 25 °C, and 35 °C). (**B**) Total exDNA degradation (%) at the end of the experiment (4 days). Different letters indicate statistically significant differences defined by *p* < 0.05 between treatments. Error bars represent the standard deviations among replicates within the treatments.

The concentration of exDNA declined rapidly after day 1 at all temperatures. The highest degradation rate (0.2547 ± 0.0536 day^−1^, *p* = 0.0177) was observed at 35 °C, in which approximately 60% of exDNA was degraded by the end of the experiment (Table 2, Figure 1B). No significant degradation was observed in control treatment (5 °C). There were statistically significant differences between 35 °C and other temperatures, including control (5 °C), starting at day 2 (F_(3,8)_ = 15.75, *p* = 0.01) (Figure 1A). The concentration of exDNA incubated at 25 °C was different from that of the control treatment only at day 3 (F_(3,8)_ = 14.66, *p* = 0.001). By the end of experiment, exDNA was degraded by up to 60% from the initial concentration in both 25 °C and 35 °C treatments. The remaining exDNA concentrations in these treatments were significantly lower (4.56 ± 0.19 × 10^11^ copies/μg DNA, *p* = 0.001, and 5.15 ± 0.38 × 10^11^ copies/μg DNA, *p* = 0.002, for 25 °C and 35 °C, respectively) than those incubated at 5 °C and 15 °C at the end of the experiment (Figure 1B).

### 3.2. Microbial Effect

**Figure 2:**
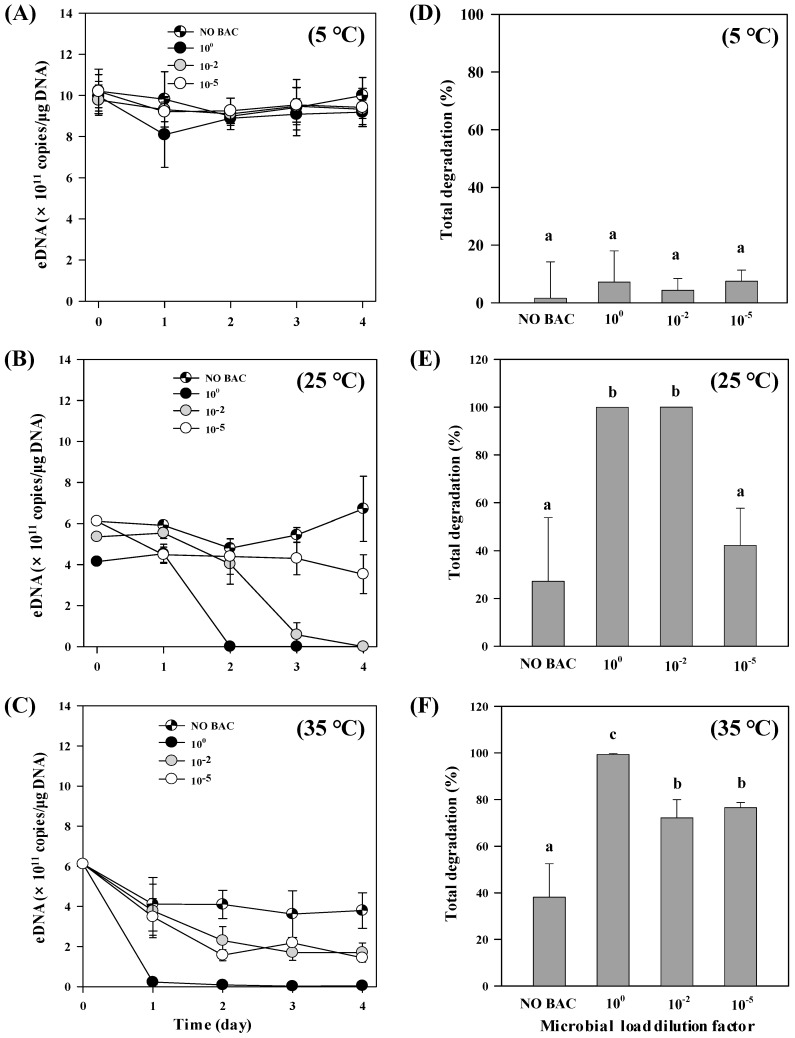
ExDNA concentration in samples exposed to different bacterial abundance and temperatures. (**A**–**C**) Changes in exDNA concentration detected under different bacterial treatments (no bacteria added and diluted bacterial solutions by 10^0^, 10^−2^, and 10^−5^-fold) at different temperatures (5 °C, 25 °C, and 35 °C). Average bacterial abundance before dilutions was 1.6 ± 0.12 × 10^7^ cells/mL. (**D**–**F**) Total exDNA degradation (%) at the end of the experiments (4 days). NO BAC: no bacteria added. Different letters indicate statistically significant differences defined by *p* < 0.05 between treatments. Error bars represent the standard deviations among replicates within treatments.

We found that higher bacterial abundance resulted in more degradation of exDNA, and microorganism activity had an interaction with high temperature, which in concert strongly accelerated the degradation to the level of the limit of detection as early as 24 h after incubation (Figure 2). The slowest degradation was observed in samples exposed to microbial activity at 5 °C, where approximately 90% of the initial exDNA remained by the end of experiment (Figure 2D). Bacterial treatments did not significantly influence the degradation of exDNA concentration at this low temperature (F_(3,20)_ = 1.542, *p* = 0.234). On the other hand, when temperature was elevated to 25 °C, the undiluted bacterial solution (1.6 ± 0.12 × 10^7^ cells/mL) caused a rapid decline in exDNA after 2 days, from there on exDNA was not detected anymore (Figure 2B). Similarly, the bacterial solution diluted by 10^−2^ (1.6 ± 0.12 × 10^5^ cells/mL) drastically reduced exDNA levels at day 3, reaching undetectable levels at the fourth day (Figure 2B). The effect of microbial activity was further enhanced by high temperature, in which exDNA degradation rate was the highest at 35 °C (Figure 2C). At this temperature, the undiluted bacterial treatment strongly reduced exDNA during the first 24 h of experiment (Figure 2C, Table 3).

### 3.3. Light Effect

**Figure 3:**
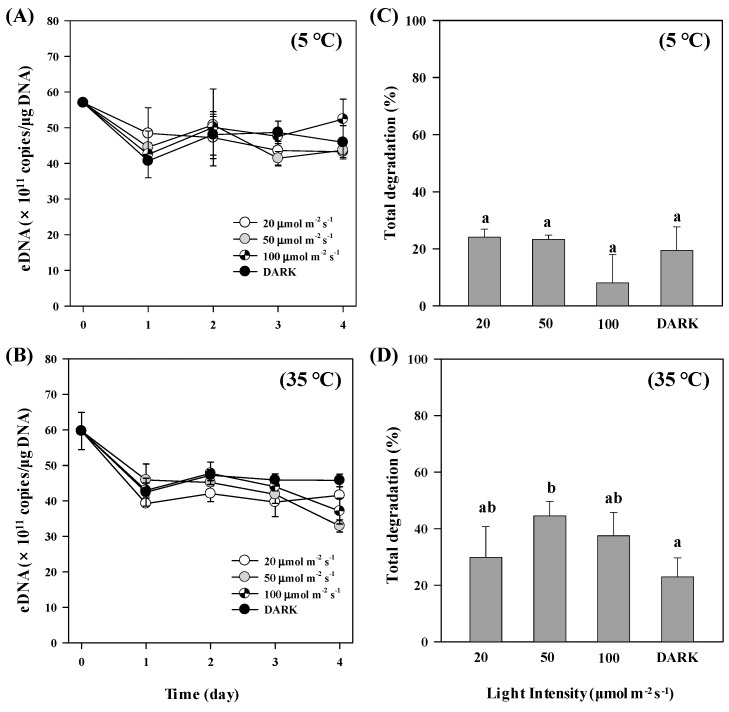
ExDNA concentration in samples exposed to different light intensity levels and temperatures. (**A**,**B**) Changes in exDNA concentration detected under different light treatments at different temperatures (5 °C and 35 °C). (**C**,**D**) Total exDNA degradation (%) at the end of the experiments (4 days). Different letters indicate statistically significant differences defined by *p* < 0.05 treatments between treatments. Error bars represent the standard deviations among replicates within treatments.

The amount of exDNA declined within 1 day in all light treatments, probably because of the bottle effect (i.e., adhesion) [46]. In all light treatments, there were no notable differences in the degradation pattern during the first 3 days of experiment. We did not find any significant differences in exDNA degradation among the different light intensity levels at 5 °C throughout the experimental period, indicating a great persistence of exDNA under these conditions (Figure 3A,C). However, at the end of the experiment at 35 °C, there was a significant difference in percentage of degraded exDNA (F_(3,8)_ = 4.075, *p* = 0.050) between samples kept in the dark and those exposed to 50 µmol m^−2^·s^−1^ (Figure 3B,D). The lowest exDNA concentration was detected in the treatment of 50 µmol m^−2^ s^−1^ under 35 °C, with the amount of 32.9 ± 1.70 × 10^11^ copies/μg DNA on the last day (Figure 3B).

### 3.4. pH Effect

**Figure 4:**
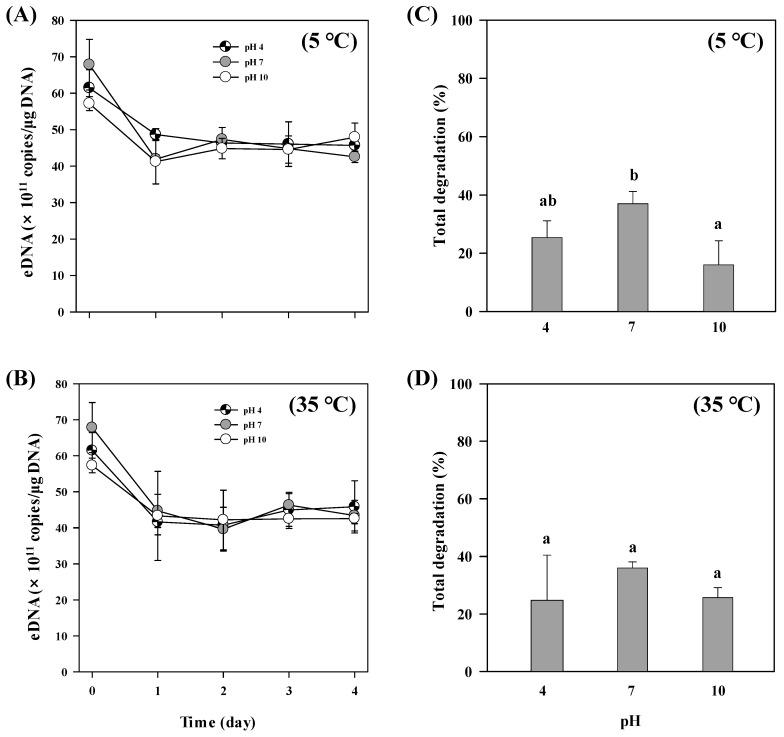
ExDNA concentration in samples exposed to different pH levels combined and temperatures. (**A**,**B**) Changes in exDNA concentration detected under different pH levels at different temperatures (5 °C and 35 °C). (**C**,**D**) Total exDNA degradation (%) at the end of the experiments (4 days). Different letters indicate statistically significant differences defined by *p* < 0.05 between treatments. Error bars represent the standard deviations among replicates within treatments.

Different pH treatments had no significant effects on the degradation rate of exDNA (Figure 4). The remaining exDNA concentration at the end of the experiment at both 5 °C (F _(2,6)_ = 2.956, *p* = 0.128) and 35 °C (F _(2,6)_ = 0.365, *p* = 0.709) did not differ (Figure 4A,B) and showed long persistence at all pH conditions. In terms of total degradation, we found that exDNA was more degraded at pH 10 than at pH 7 when incubated at 5 °C (F _(2,8)_ = 8.341, *p* = 0.019) (Figure 4C), in which 37% of the initial exDNA was degraded.

## 4. Discussion

This study evaluated how different levels of biotic (microbial activity) and abiotic factors (temperature, light, and pH) affect the persistence of exDNA from a eutrophic freshwater system. Our results of exDNA degradation followed an exponential decay pattern, which is similar to that of iDNA recorded in previous studies [28,29,31,47]. Observations under controlled conditions resulted in 7–99% degradation of the initial amount of exDNA after 4 days. The degradation rates 0.0931–3.2706 day^−1^ fell within the estimated rates in other studies (Table 4).

The amount of exDNA preserved in the sediment as a whole is known to be large because it is pooled and accumulated in the sediment [37,48]. However, the exDNA content varies widely over the whole sediment due to its spatial heterogeneity. One past study reported that PCR amplification on DNA recovered from marine sediment did not contain targeted prokaryotic 16S rDNA genes even though the purity of exDNA recovered was sufficiently high [24]. After isolation from the lake sediment and quantification by qPCR using specific primers, we confirmed that the amount of cyanobacterial exDNA were not that high in the total exDNA obtained, which led us to add exDNA from *Anabaena variabilis* culture. Based on our results, we suspect that the rapid degradation of exDNA under various environmental parameters in the natural sediment might be attributable to the small quantity of exDNA found in our samples.

Previous studies observed a strong influence of temperature on the degradation of eDNA, where a slow degradation rate was often associated with low temperature and high temperature tends to enhance eDNA degradation [29,30,31,35]. Accordingly, we found that incubation at room temperature or higher promotes the degradation of exDNA at an exponential rate. The sudden decline at 25 °C that occurred between days 3 and 4 relative to 35 °C likely happened because of bacterial contamination during extraction or sampling. Such a minor cross-contamination is unfavorable, but it is hard to avoid as observed in similar eDNA degradation studies, where the movement of small water droplets by aeration occurs even within samples separated by 0.2 m [28,29]. Contamination involving DNA extraction kits and their reagents have also been reported as a challenge for low biomass studies and significantly influence the result of microbiota studies [49]. Therefore, the non-template negative control should be considered to monitor the probability of cross-contamination across all samples and thus improve the accuracy of results. In our experiment, we only assigned non-treatment negative controls, in which we expected long persistence of exDNA throughout the test. Even though the possibility of bacterial cross-contamination cannot be ruled out in our treatments, it should not affect our results of temperature effects because we used specific primers that target only cyanobacterial 16S rDNA.

In the microbial activity experiments, we evaluated the degradation effect by interacting various levels of bacterial concentration under different temperatures to mimic low to high microbial activity. To the best of our knowledge, this is the first study that simultaneously combined bacterial levels and temperature to observe microbial activity effects on eDNA degradation. Our findings provide strong evidence that high temperature associated with high bacterial abundance can accelerate the degradation of exDNA even within 24 h. This result indicates that microbial effect leads to a faster degradation of exDNA than that of iDNA reported in previous studies, probably owing to its vulnerability to environmental factors. The significance of microbial activity as a strong driver of eDNA degradation has been acknowledged previously, even though its role seems to be complex and varied under interaction with other environmental factors [28,30,35]. For example, the relative decay of eDNA sourced from bighead carp biowaste and silver carp sperm in the combined treatments including microbial loads, temperature, and pH resulted in 90% loss of eDNA within only 5 days [30]. On the other hand, any bacterial abundance levels in combination with different temperatures did not significantly affect common carp eDNA degradation [31]. Microbial activity is well recognized to be temperature-dependent [50]. However, one study conducted using common carp eDNA revealed that bacterial enzymatic activity did not have significant effects on eDNA degradation, although higher bacterial abundance was positively correlated with higher water temperature [31]. In contrast with our experiment, this study only allowed a single concentration of bacteria to interact with three levels of temperature (10 °C, 20 °C, and 30 °C). Our study independently controlled known levels of bacterial concentration (1.6 ± 0.12 × 10^7^ cells/mL) ranging from high to low concentration through serial dilution (10^0^, 10^−2^ and 10^−5^ fold) and different temperatures (5 °C, 25 °C, and 35 °C) to create different levels of microbial activity. Increased microbial activity is likely to release more DNase that ultimately degrade and utilize exDNA as a source of carbon, nitrogen, phosphorus, and nucleic acid precursors [14,51].

Because of the depletion of the stratospheric ozone layer, UV-B radiation can penetrate into the water column and induce eDNA degradation by disrupting DNA base-pairs bonds [52]. In contrast with our expectation, different levels of light intensity had little significant effect on the degradation rate of exDNA in our study. This may be explained by the fact that light intensity levels selected in this experiment might be lower than those expected to influence exDNA degradation. Most of previous eDNA degradation studies examined the effect of light using UV radiation. One study observed in an outdoor experiment that the effect of solar radiation levels had little effect on the persistence of eDNA among the treatments including sun exposure, 20% UV exposure, and dark treatments, concluding that degradation occurs even in the absence of light [47]. Surprisingly, in a recent study, UV radiation, regardless of UVA or UVB radiation type, has been found to have no effect on eDNA detectability [33]. By co-varying light intensity and temperature to simulate natural conditions, we found a significant difference only by the end of experiment at 35 °C treatment compared to 5 °C treatment (Figure 3A–D). This result implies that temperature would be a stronger driving factor than light intensity for exDNA degradation. A similar degradation experiment using a full-factorial design, involving UVB, temperature, and pH effects, conducted by [29] showed that while UVB and temperature had positive effect on degradation of eDNA independently, opposite effects occurred when interaction with other factors were included. Nevertheless, in a real ecosystem, we must consider a potential effect of UV on exDNA degradation, which may depend on UV transparency of the water column, being altered by geographic characteristics and the concentration of dissolved organic carbon, organic matter, and humic acids [53]. Therefore, further studies should include a broader range of light intensity along with factors that can affect its penetrability into the water column, in order to understand the total light effect on the decay rate of exDNA.

Past studies reported DNA hydrolysis is favored under acidic conditions [54,55]. However, it was found that the higher eDNA degradation rate in pH 4 was because of interactions with other variables, and that the acidic condition itself did not affect degradation [29]. Across an acid-base gradient of stream mesocosms, the decay rate of lotic multispecies eDNA was accelerated to non-detectable levels within 2 days in acidic environments [32]. The effects of pH on eDNA persistence might be complex. In another study, applying a fairly narrow range of pH near 7.0, eDNA degradation rate was higher under pH 8 than that under pH 6.5 [30]. In practice, the effect of pH on eDNA degradation under laboratory-controlled conditions may not be always consistent with that observed in real ecosystems. In a recent study, the exposure of eDNA to different pH levels revealed that eDNA yield was highest under acidic conditions [56]. The difference between the yields of two experiments in that study indicated that pH effects were complex and might be related to other factors such as adsorption effects during filtration process and the type of eDNA filtered. Our study used exDNA and the pH effect was only significant when comparing pH 10 to pH 7 in terms of total degradation (Figure 4C). Nevertheless, there was no pH interaction with temperature at all levels in relation to the degradation rate of exDNA (Figure 4A,B). Due to small volumes in our treatment, we were concerned about pH changes in the samples (i.e., dilution effect) when placing a water tray to avoid evaporation that might happen inside a 35 °C incubator. Therefore, we took precautionary steps by using microplates with covers and placing the water tray 30 cm below the microplates in the incubator chambers. Nonetheless, we suspect that pH effects in our experiment might be different from those in other studies because of the limitation of the small volumes used in our treatment.

Other variables may affect eDNA persistence and it becomes uncertain if spatiotemporal context is also taken into account. A previous comparative study between water samples and sediment samples demonstrated that sedimentary eDNA was 8–1800 times more concentrated than that in water samples and indicated the sediment might be the key source of long-lasting genetic material for species detection based on eDNA approach [25]. Sediment-derived exDNA, such as that used in our study, may be associated with inhibitory properties of sediment and preserved in a stable form. Sediment-associated DNA binds to clay minerals, sand, and humic substances, which may prolong DNA persistence [37,57]. Thus, certain environmental conditions might reduce the degradation of exDNA recovered from the sediment rather than from water column. However, the high level of bacterial abundance in the sediment could affect degradation of exDNA accumulated over a long period of time no matter how its binding properties protected them from nuclease degradation. Bacterial abundance found in our sediments samples was 1.6 ± 0.12 × 10^7^ cells/mL or 3.2 ± 0.12 × 10^9^ cells/g of sediment and within the range of those measured in past studies which was between 10^7^ cells and 10^9^ cells/g of sediment [17,19]. Based on our result, we assume that in real ecosystem, the high bacterial abundance associated with relevant physiochemical factors can accelerate the degradation of exDNA and affect the target species detectability over time.

## 5. Conclusions

This study demonstrated that different environmental conditions can affect exDNA degradation rate. Among all factors tested, temperature and microbial activity showed the strongest positive effects on degradation rate of exDNA within a short period of time. Even though light intensity and pH did not significantly influence exDNA degradation rate, combined treatment of temperature with these factors accelerated the exponential decay. We expect that interaction between various environmental factors under natural settings could reduce exDNA detectability over time. Using controlled conditions simulating a range of natural freshwater ecosystem parameters, our results provide a new insight on the degradation rate of exDNA, whose fast degradation occurs within 1 or 2 days. Our results provide evidence that supports the understanding of factors underlying degradation of extracellular eDNA and might be useful in further research on extracellular eDNA detectability in natural freshwater environments.

## Figures and Tables

**Table 1:** The summary of the treatments and their interacting factors. Other treatments than light intensity were conducted in the dark and all treatments were run separately in triplicate over the 4 days.

Treatment	Levels	Interacting Factors
Temperature	5 °C 15 °C 25 °C 35 °C	None
Bacterial activity	No Bacteria 1.6 ± 0.12 × 10^7^ cells/mL (10^0^ fold) 1.6 ± 0.12 × 10^5^ cells/mL (10^−2^ fold) 1.6 ± 0.12 × 10^2^ cells/mL (10^−5^ fold)	Temperature (5 °C, 25 °C, 35 °C)
pH	pH 4 pH 7 pH 10	Temperature (5 °C, 35 °C)
Light intensity	Dark 20 μmol m^−2^ s^−1^ 50 μmol m^−2^ s^−1^ 100 μmol m^−2^ s^−1^	Temperature (5 °C, 35 °C)

**Table 2:** Degradation rates of exDNA under different temperature treatments (5 °C, 15 °C, 25 °C, and 35 °C) during incubation for 4 days. Degradation rates were estimated by fitting the number of copies of each set of DNA to an exponential decay curve. SE: standard error.

Temperature (°C)	Degradation Rate (*r*) (day^−1^)	SE	*p-*Value
5	0.0692	0.0298	0.0353
15	0.1201	0.0339	0.0383
25	0.1942	0.0599	0.0479
35	0.2547	0.0536	0.0177

**Table 3:** Degradation rates of exDNA under different bacterial treatments at 35 °C during incubation for 4 days. Three serial dilutions of 10^0^, 10^−2^, 10^−5^ fold were made from the prepared bacterial solution. Average bacterial abundance in solution before dilution was 1.6 ± 0.12 × 10^7^ cells/mL. Degradation rates were estimated by fitting the number of copies of each set of DNA to an exponential decay curve. SE: standard error.

Dilution Factor	Bacterial Concentration	Degradation Rate (*r*) (day^−1^)	SE	*p-*Value
None	0	0.1297	0.0472	0.0709
10^0^	1.6 ± 0.12 × 10^7^ cells/mL	3.2706	0.2178	0.0006
10^−^^2^	1.6 ± 0.12 × 10^5^ cells/mL	0.4112	0.0505	0.0039
10^−5^	1.6 ± 0.12 × 10^2^ cells/mL	0.4513	0.0987	0.0196

**Table 4:** Comparison of eDNA decay rates among different eDNA types, sources, and environmental factors. * indicate the factors that had a significant effect on eDNA degradation in the respective study. eDNA: Environmental DNA, UV-B: Ultraviolet B light, OECD: Organization for Economic Co-operation and Development.

Reference	eDNA Type	Source	Environmental Factor	Decay Rate, (*r*) (day^−1^)	
This study	Extracellular	Sediment sample Cyanobacterium *Anabaena variabilis*	Temperature *, microbial activity *, pH, light intensity	0.0931–3.2706	
[32]	Intracellular	Crustacean *Daphnia magna*	pH *, temperature, microbial activity, total dissolved nitrogen	Water derived	6.552–23.568
		Mayfly *Ephemera danica*			
				Biofilm derived	1.176–17.256
		Eel *Anguilla anguilla*			
[31]	Intracellular	Ayu sweetfish *Plecoglossus altivelis altivelis*	Temperature *, microbial abundance	0.48–7.2	
		Common carp *Cyprinus carpio*			
[35]	Intracellular	Common carp *Cyprinus carpio*	Temperature *, trophic state *	0.35–2.42	
[29]	Intracellular	American bullfrog *Lithobates catesbeianus*	UV-B *, temperature *, pH	0.243	
[28]	Intracellular	Common carp *Cyprinus carpio*	Microbial community *, pH	2.52	
[27]	Extracellular	Sediment and water samples	Based on simplified OECD endurance test	0.009–0.133	
